# Continuity of Care During COVID-19 Lockdown: A Survey on Stakeholders' Experience With Telerehabilitation

**DOI:** 10.3389/fneur.2020.617276

**Published:** 2021-01-13

**Authors:** Carla Assenza, Hilenia Catania, Clementina Antenore, Tiziana Gobbetti, Paola Gentili, Stefano Paolucci, Daniela Morelli

**Affiliations:** ^1^Neurorehabilitation Outpatient Department, Santa Lucia Foundation (Scientific Institute for Research and Health Care), Rome, Italy; ^2^Clinical Laboratory of Experimental Neurorehabilitation, Santa Lucia Foundation (Scientific Institute for Research and Health Care), Rome, Italy

**Keywords:** continuity of care, COVID-19, telerehabilitation, caregivers, rehabilitation professionals, perception, satisfaction

## Abstract

**Objective:** To explore professionals', adult patients', and children's caregivers' perception and satisfaction with telerehabilitation during COVID-19 lockdown.

**Design:** An observational transversal study on a web-based survey was conducted in order to explore participants' perception and satisfaction of telerehabilitation during COVID-19 lockdown.

**Setting:** The study was conducted at our Outpatient Neurorehabilitation Service.

**Subjects:** All rehabilitation professionals, adult patients, and children's caregivers who accepted telerehabilitation were recruited.

**Interventions:** Participants had to respond to the Client Satisfaction Questionnaire-8 and to a purpose-built questionnaire on their perception and satisfaction of the service provided.

**Main Measures:** Data were analyzed by qualitative statistics and logistic regression models.

**Results:** All 144 caregivers, 25 adult patients, and 50 professionals reported a medium-high level of perception and a high level of satisfaction. Results showed a correlation among caregivers of children aged 0–3 and feeling overwhelmed with remote care (OR = 3.27), a low perception of telerehabilitation for enhancing goals (OR = 6.51), and a high perception of feeling helped in organizing daily activity (OR = 2.96). For caregivers of children aged over 6 years, changes in the therapy plan were related to a low perception of feeling in line with the in-person therapy (OR = 2.61 and OR = 9.61) and a low satisfaction (OR = 5.54 and OR = 4.97). Changes in therapy were related to concern (OR = 4.20). Caregivers under 40 and professionals showed a high probability to perceive telerehabilitation as supportive (OR = 2.27 and OR = 5.68). Level of experience with remote media was shown to influence perception and satisfaction.

**Interpretation:** Telerehabilitation can be a useful practice both during a health emergency and in addition to in-presence therapy.

## Introduction

During public health crises, as in the COVID-19 pandemic, telemedicine can be a viable opportunity for reducing risk of infection while offering solutions to the constant demands of care.

Evidence on the merits of this service is provided by NATO, which, during various crises, developed a multinational telemedicine system deployed with military forces (1). Another example is China, which, during the SARS pandemic, began to examine telemedicine and integrated medical systems for future use in similar circumstances (2).

International health agencies such as WHO are fundamental for large-scale deployment of telemedicine services. Embedding its practice into routine service delivery with guidelines is the most effective way to prove its important role in health care.

The primary purpose of this study was to analyze the perception of remote rehabilitation during lockdown by adult patients, children's caregivers, and rehabilitation professionals and to verify their level of satisfaction with the service provided. Possible individual factors influencing stakeholders' perception of telerehabilitation were examined by a multivariate analysis. Treatment effectiveness was not investigated in this study. As a survey study, it should be interpreted with caution and findings cannot be generalized but rather be considered as suggestions. In addition, as a monocentric study, results may be related to the service provided by our department rather than to telerehabilitation itself.

## Materials and Methods

### Study Design

An observational transversal study on a web-based survey was conducted in order to explore participants' perception and satisfaction of telerehabilitation during COVID-19 lockdown.

The study was approved by the Independent Ethic Committee of the Research Institute of the Santa Lucia Foundation.

Therapists, adult patients, and children caregivers in charge were asked to complete a two-section survey on their perception and satisfaction of an in-home video telerehabilitation approach. The survey included two sections: an informant section and a section assessing the perception of remote rehabilitation. Adult patients and children's caregivers' survey included an additional section on the level of satisfaction with the service provided, the Client Satisfaction Questionnaire-8 (CSQ-8) (3–5).

An introductory explanation of the purpose of the questionnaire preceded the survey. Three questionnaires in Italian were developed, validated, and administered.

A review of the literature was performed in order to detect questionnaires that evaluate telerehabilitation. Members of the consensus panel, a psychologist, two physicians, and a physical therapist, generated and outlined the items. A draft was assessed through a validation procedure and then tested in study samples. Ten therapists, 10 caregivers, and 10 patients were recruited to assess relevance of draft questions.

The first version consisted of 62 items for all three study groups. A consensus panel rated the contents and purpose of each item and selected three 25-item closed question questionnaires. Relevance and clarity of each statement were then assessed by experts on a four-point Likert-type scale (6).

An item-level content validity index (I-CVI) was computed for relevance and simplicity. A score of 0.78 was selected as the threshold for an acceptable I-CVI (7, 8). A scale-level content validity index was calculated as the average across items' I-CVI (S-CVI/Ave) and as the proportion of items that all experts rated as relevant or simple (S-CVI/UA, scale-level content validity index universal agreement), with selected thresholds of 0.90 and 0.80 for an acceptable S-CVI/Ave and S-CVI/UA, respectively (7, 9). The items were revised, thus generating a 20-item questionnaire for therapists and 15-item ones each for caregivers and for patients.

Each question was assigned with a score (0–5 points). The sum of the scores ranges from a minimum to a maximum score equivalent to the worst and best perception of telerehabilitation during lockdown.

The CSQ-8 is a self-administered eight-item standardized questionnaire, developed by Larsen et al., aimed to assess the client/patient satisfaction with services provided (3–5). It is a four-point Likert scale that estimates several aspects of a service provision. For each item, four scored answers are possible. The sum of all items is the total score ranging from a minimum of 8 to a maximum of 32, so that the higher the score, the higher the level of satisfaction. The CSQ-8 has previously been used to measure the level of satisfaction of children's caregivers and with a remote rehabilitation service (10, 11). A written formal license agreement to use the Italian version of the scale on an electronic platform was provided by the copyright holder before starting our study.

### Setting

Due to lockdown, outpatient rehabilitation services were suddenly interrupted. In response to this situation, a prompt adaptation of delivery modes in order to support ongoing services was called for. Remote delivery of care seemed to be the ideal approach for providing access to therapy sessions, although not typically used in the department. The service was proposed to both children and adults in charge. Professionals were involved in initial contacts of patients and families in order to collect information on technical, personal resources, and permission for remote treatment. Despite the initial difficulties due to unavailability of technical equipment, remote care began within a week after lockdown. Team members adapted some aspects of previous in-person therapy plans in order to remotely continue progress toward goals.

Treatment plans included physical, speech, occupational, and cognitive–behavioral therapy for the group of children, and neuropsychological therapy and psychological support to adolescents, adult patients, and families. Sessions were conducted from the workplace to the patient's home, via tablet, smartphone, or PC using video meeting systems such as Google Meet or Skype. Activity did not follow a standardized scheme but was individualized for each patient based on his/her clinical features and type of device used. Caregiver mediation depended on the patient's age, level of cognitive function, type, severity of functional impairment, and level of task difficulty. Efforts were made to ensure treatment was provided to the patient by the same professionals before lockdown. Number of sessions, treatment type, and duration (50 min) were in line with the original Individual Rehabilitation Plan. Research participation consent forms were emailed to patients or to minors' parents or legal guardians, guaranteeing anonymity. Remote treatment began in March while the invite to complete the online survey hosted by Google Forms was sent in May, after 2 months of treatment.

### Participants

All professionals, adult patients, and children's caregivers of the Outpatient Neurorehabilitation Service were recruited. At the beginning of lockdown, 362 patients, comprising 270 children (primarily with cerebral palsy, genetic disorders, neuromuscular diseases, and prematurity) and 92 adults with complex disorders (primarily stroke, acute brain injury, spinal cord injury, Parkinson disease, and multiple sclerosis), were in charge. Consent was given by 265 families of minors and by 48 adult patients. Only the professionals that worked for at least 1 month during the project were considered qualified.

### Variables

Each survey included a first section for recording several variables, namely, demographic and other personal information. The assessed variables for rehabilitators were as follows: age (21–30 years, 31–40 years, 41–60 years, or >60 years), professional position (Physical, Neurodevelopmental, Speech, Occupational Therapists, and Psychologist), years of work experience (<5 years, 5–10 years, 10–20 years, or >20 years), remote media skill level (none, low, sufficient, high, or very high), and previous experience with remote care (yes or none). Patients were asked about their age (<20 years, 21–40 years, 41–60 years, or >60 years), number of therapy sessions (2, 4–6, or >6), rehabilitation plan and type of therapies, level of familiarity with remote media (none, low, sufficient, high, or very high), need for assistance to perform exercises (yes or no), and support availability (yes or none). Information regarding caregivers and their children included sex of caregiver (male or female), age of caregiver (<40 years, >40 years, or not reported), age of the child (0–3 years, 4–6 years, >6 years, or not reported), rehabilitation plan and therapies performed during remote mode (rehabilitation programs respected, modified, or information not reported), and caregiver's experience with remote media (yes or none). This information served to define the sample and to analyze possible correlations with different levels of perception and satisfaction of remote treatment.

### Data Analysis and Statistics

Research data were downloaded from Google Forms platform, exported to a Microsoft Excel spreadsheet for data analysis. Both qualitative and quantitative statistical analysis were performed (12–14).

Sample characteristics were expressed in percentages (%), while data on the perception and satisfaction statements were analyzed by a descriptive qualitative method and by median and standard deviation measures.

Forward, stepwise, and Wald logistic regressions were performed in order to investigate the correlations between the examined variables and the level of perception and satisfaction expressed, thus allowing us to hypothesize how the experience of telerehabilitation during lockdown was influenced by demographic, personal factors, or therapy plan.

The logistic model was not applied due to the small sample of adult participants.

Dependent variables were as follows: level of agreement of statements expressed by participants (not at all, little, enough, highly, or strongly) and level of satisfaction (quite dissatisfied, indifferent or mildly dissatisfied, mostly satisfied, or very satisfied).

Independent variables (0 = if absent and 1 = if present) were different for the two groups. For the caregivers' group, demographic and personal information were considered. Given the number of health professionals participating in the study, a smaller number of independent variables were examined: age (<40/>40 years), professional role, type of patient treated (adults/children), years of work experience (<10 years/>10 years), skill with remote media (yes/not), and previous experience with remote care delivery (yes/not).

Data analyses were performed using the SPSS 17.0 Statistical Package for Social Sciences.

## Results

Participation rate for professionals was 100%. Only 2 physiotherapists and 1 speech therapist out of the 53 professionals in service during March did not meet inclusion criteria and were excluded from the study; 50 took part in the study. In the adult group of patients and in the group of children's caregivers, participation rate to the survey was, respectively, 58.06% (*n* = 25) and 67.56% (*n* = 144).

Data from an online survey on 25 adult patients, 144 children's caregivers, and 50 professionals were collected and analyzed.

**Figure 1:**
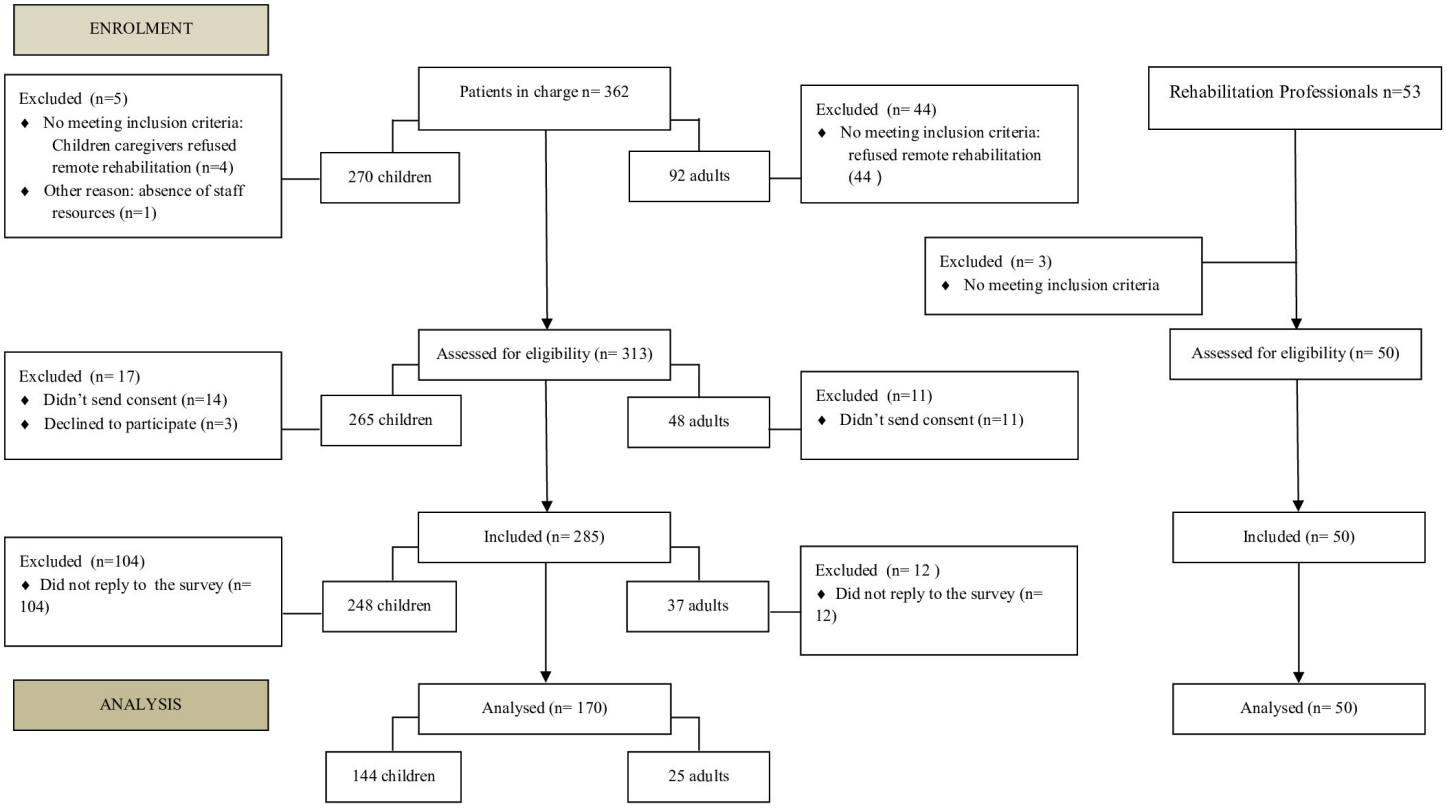
Flow chart of participants.

Process leading to the final number of participants and selection stages are shown in Figure 1.

The professionals' sample included 20 physical therapists (40%), 12 speech therapists (18%), 9 neurodevelopmental therapists (24%), 4 occupational therapists (10%), and 5 psychologists (8%).

Eighteen were aged between 41 and 60 years (36%), 14 were under 30 years of age (28%), 13 were aged between 31 and 40 (26%), and 5 were over 60 years of age (10%). Thirty-four had a work experience of over 10 years (68%); 13 (26%) reported no familiarity with remote media and 18 (36%) reported previous remote treatment experiences.

The adult sample primarily consisted of patients over the age of 60 (44%); 17 of them (65.4%) underwent biweekly treatment; mainly physical therapy, both before (80%) and after (72%) lockdown; 9 (36%) declared no confidence with remote media; 10 (40%) needed assistance to perform the proposed exercises; and 2 (7.7%) reported difficulties in availability of caregiver assistance.

As for the 144 caregivers, 102 were females (70.83%) and 78 (54.16%) were above 40 years of age. The children's sample consisted of 48 (33.33%) aged from 0 to 3 years, 40 (27.77%) aged from 4 to 6 years, and 50 (34.72%) above 6 years of age. Six (4.17%) caregivers did not reveal the age of their child. As for the therapy plan, 127 caregivers (88.19%) reported continuity of rehabilitation plan, while 16 caregivers (11.11%) referred changes. Eighty-five caregivers (59.03%) reported no familiarity with remote media. One caregiver did not provide personal information.

Children's caregivers sample obtained a mean score of 53.27 (SD 10.60) on the perception questionnaire. This score falls in a medium-high range considering 15 as the worst perception and 75 as the best perception.

**Figure 2:**
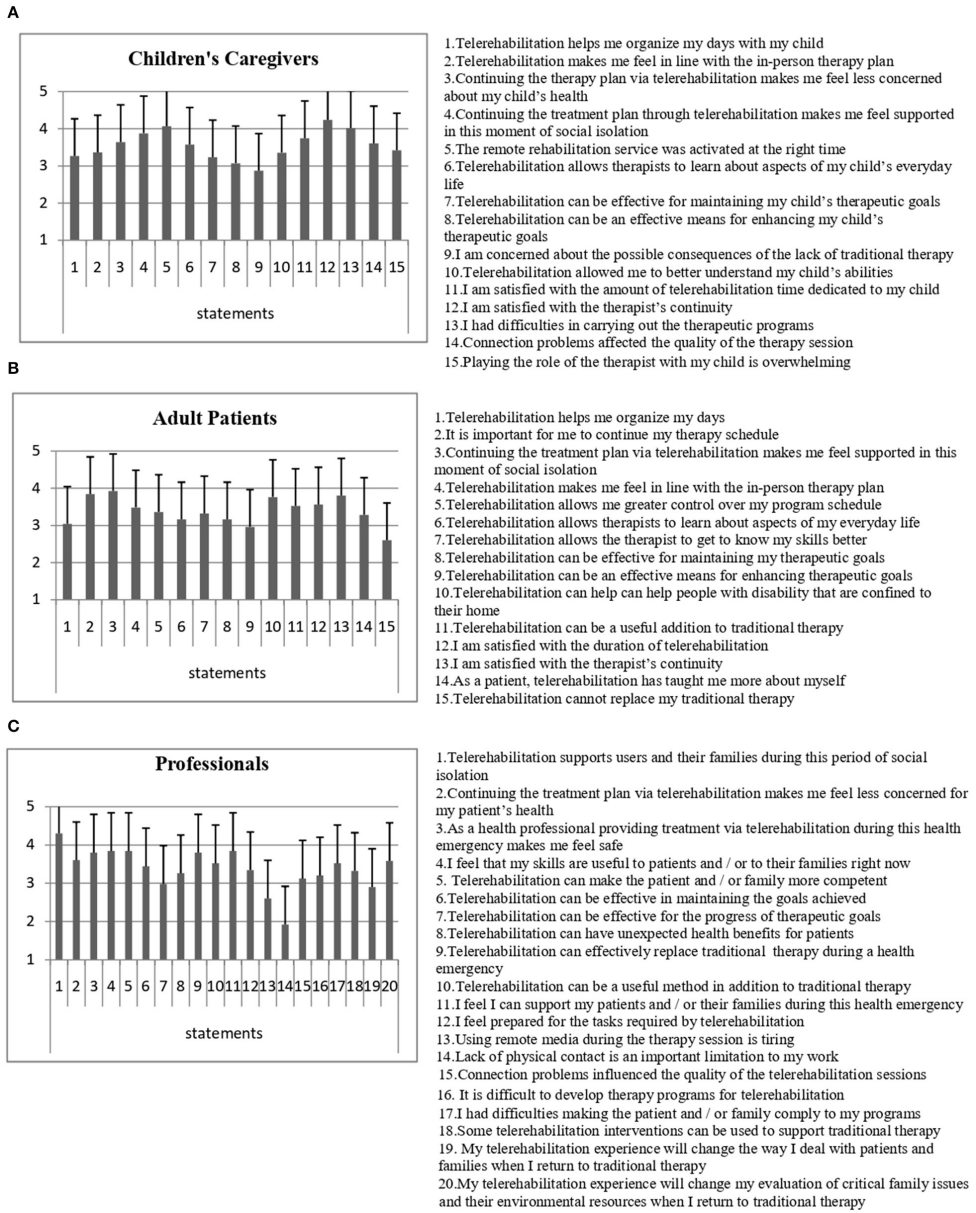
**(A)** Results of children's caregivers' perception questionnaire. **(B)** Results of adult patients' perception questionnaire. **(C)** Results of rehabilitation professionals' perception questionnaire. Means and SD values are represented.

Figure 2A shows the mean scores and the standard deviations of the 15 statement responses. These results must be interpreted taking into account that, for each statement, score 1 represents the worst perception and score 5 represents the best perception. Some were negative statements; the graph shows the perception values already converted into the five-point Likert scale.

The mean total score of the patient's perceptions questionnaire was 50.76 (SD 8.23) (Figure 2B).

Results of the professionals' sample (Figure 2C) show an average total score of 67.66 (SD 8.57) where 100 corresponds to the best and 20 corresponds to the worst perception.

**Figure 3:**
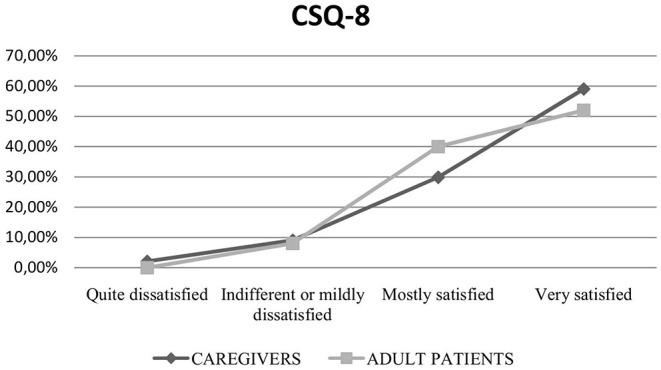
Results of the CSQ-8 of children's caregivers' and adult patients' samples.

The results of the CSQ-8 questionnaire showed a mean score of 26.8 (SD 4) corresponding to a medium-high level of satisfaction with the telerehabilitation service, in both adults and children's caregivers' sample (Figure 3).

Tables 1, 2 show results of the regression model selection and estimated changes in log odds and related standard errors. Results showed a correlation among caregivers of children aged 0–3 and feeling overwhelmed with remote care (OR = 3.27); a low perception of telerehabilitation for enhancing goals (OR = 6.51) and a high perception of feeling helped in organizing daily activity (OR = 2.96). For caregivers of children aged over 6 years, changes in the therapy plan were related to a low perception of feeling in line with the in-person therapy (OR = 2.61 and OR = 9.61) and a low satisfaction (OR = 5.54 and OR = 4.97). Changes in therapy were related to concern (OR = 4.20). Caregivers under 40 and professionals showed a high probability to perceive telerehabilitation as supportive (OR = 2.27 and OR = 5.68). Level of experience with remote media was shown to influence perception and satisfaction.

**Table 1:** Results of forward stepwise logistic regression relative to the children's caregivers' sample.

**Independent variables**	**Dependent variables**	***B***	**SE**	***p***	**OR**	**95% CI**	
Child aged 0–3 years	High level of agreement with the sentence: “*Telerehabilitation helps me organize my days with my child”*	1.08	0.41	0.008	2.96	1.32	6.64
	Very low level of agreement with the sentence: “*Telerehabilitation can be effective means for enhancing my child's therapeutic goals”*	1.87	0.75	0.013	6.51	1.47	28.75
	High level of agreement with the sentence: “*Playing the role of the therapist with my child is overwhelming”*	1.18	0.50	0.019	3.27	1.21	8.78
Rehabilitation programs modified	Very low level of agreement with the sentence: “*Telerehabilitation makes me feel in line with the in-person therapy plan”*	2.26	0.86	0.009	9.61	1.75	52.59
	Low level of agreement with the sentence: “*Continuing the treatment plan via telerehabilitation makes me feel less concerned about my child's health”*	2.76	1.04	0.008	15.83	2.03	122.9
	Very high level of agreement with the sentence: “*I am concerned about the possible consequences of the lack of traditional therapy”*	1.43	0.58	0.014	4.20	1.34	13.12
	Very low level of agreement with the sentence: “*Telerehabilitation has allowed me to better understand my child's ability”*	1.73	0.78	0.027	5.67	1.21	26.51
	Low level of satisfaction	1.59	0.80	0.048	4.97	1.01	23.80
Child aged >6 years	Low level of agreement with the sentence: “*Telerehabilitation makes me feel in line with the in-person therapy plan”*	0.96	0.45	0.036	2.61	1.06	6.43
	Low level of satisfaction	1.71	0.68	0.013	5.54	1.14	21.31
No experience with remote media	Very high level of satisfaction	−0.76	0.34	0.028	0.46	0.23	0.92
Caregiver aged <40 years	Very high level of agreement with the sentence: “*Continuing the treatment plan through telerehabilitation makes me feel supported in this moment of social isolation”*	8.24	0.37	0.028	2.27	1.09	4.75

*Only significant independent variables are shown (B, regression coefficient; SE, standard error; OR, odds ratio; CI, confidence interval)*.

**Table 2:** Results of forward stepwise logistic regression relative to the Rehabilitation Professionals' sample.

**Independent variables**	**Dependent variable**	***B***	**SE**	***p***	**OR**	**95% CI**	
NDDs/PTs/OTs	High level of agreement with the sentence: “*Telerehabilitation is important to support users and their families in this time of social isolation*”	1.74	0.67	0.01	5.68	1.51	21.42
	High level of agreement with the sentence: “*Telerehabilitation can be effective in maintaining the goals achieved”*	1.66	0.79	0.036	5.28	1.14	25.09
No experience with delivering care remotely	Very high level of agreement with the sentence: “*Telerehabilitation can be a suitable model of intervention to replace traditional therapy in emergency situations”*	2.12	1.01	0.037	8.30	1.14	60.53
Previous experience with delivering care remotely	High level of agreement with the sentence: “*Telerehabilitation can be useful as a method in addition to traditional therapy”*	2.25	0.80	0.005	9.52	1.96	46.15
Confidence with remote media	High level of agreement with the sentence: “*Telerehabilitation can make the patient and/or the family more competent”*	2.00	.093	0.032	7.43	1.19	43.39
Age <40 years		2.26	1.07	0.035	9.58	1.17	78.76

*Only significant independent variables are shown (B, regression coefficient; SE, standard error; OR, odds ratio; CI, confidence interval; NDDs, neurodevelopmental therapists; PTs, physical therapists; OTs, occupational therapists; ST, speech therapists; PSY, Psychologists)*.

## Discussion

Lockdown restriction measures imposed by the COVID-19 diffusion included the interruption of nonessential services such as rehabilitation services for non-urgent cases resulting in an ethical dilemma, regarding the right of access to care and of continuity of care (15–17). As an answer to this, telerehabilitation was proposed to all patients in charge at our rehabilitation outpatient department.

### Perception and Satisfaction of Telerehabilitation by Caregivers of Disabled Children

Results of the perception questionnaire revealed an overall medium-high level of positive perception of remote treatment; thus, telerehabilitation could be suggested as an alternative method during a health emergency. Caregivers expressed a good perception of the timely service activation, the specialist's constant presence and the feasibility of the required task. These results are supported by the efforts made to quickly activate the service, to ensure continuity of care by the same therapists and by guaranteeing the treatment plan (88.19%). Good perception of the feasibility of the required tasks may depend on the patient-designed treatment conducted by the same therapist who knew the child and his/her family.

Caregiver's concern about the possible consequences of interrupting the in-person therapy is probably due to parental anxieties and worries about their child's condition (18–21). Parents of children with complex needs often feel they do not have enough resources to take care of their children. In addition to this, during lockdown, parents had to play many different roles, including that of therapists, increasing their sense of inadequacy and inducing fears about the possible consequences of poor practice (22–24). Based on the logit regression, playing the role of therapists was perceived as overwhelming by parents of children aged 0–3 years. This burden could be aggravated by the attention and care required by babies and toddlers in general and by fear of COVID-19 (25, 26).

Logit regression reported a relationship between a low perception of the effectiveness of telerehabilitation in the enhancement of therapeutic goals and parents of 0- to 3-year-old children.

Parents are aware that before the age of 2, the brain is still developing and there is a critical developmental window in which an early intervention may influence brain development, and this knowledge could explain results (19, 27).

The same group of parents showed a correlation with the statement that “telerehabilitation helps them to organize the daily schedule.” Mothers of disabled children seem to have a higher level of stress induced by daily routines (28). Lockdown caused changes in daily schedule, increasing parents' stress level (26, 29). Remote sessions might have allowed parents to maintain a fixed appointment within an uncertain family routine.

Logistic model also showed a higher probability of caregivers of children over 6 years of age to express a low level of agreement with the statement that “telerehabilitation makes them feel in line with the in-person therapy plan.” Changes in therapeutic goals, expectations, and concerns vary with child's age and clinical condition.

Parents of children with cerebral palsy, under the age of 2 and aged between 2 and 4, are more concerned about motor skills while parents of children aged over 6 years are mainly concerned about worsening of clinical conditions (30). In-person therapy suspension may have increased parent's worries about the child's abilities worsening and could have led them to perceive telerehabilitation as an interruption of continuity of care and to express a low level of satisfaction.

The logistic regression model revealed a correlation between the group of caregivers of children whose amount and type of therapy was not guaranteed and a low level of satisfaction with the telerehabilitation service, confirming that the continuity of care and of therapists influences parents' satisfaction of therapy intervention (31). Moreover, results showed further correlations among this group of caregivers and statements regarding “not feeling in line with the in-person therapy” and being “concerned about the possible consequences of the lack of traditional therapy.” These results are supported by the interruption of the treatment plan, by the increased concerns, and by the changes in the treatment plan during telerehabilitation.

Logistic regression showed a negative relation between the group of caregivers who expressed “no experience with remote media” and a very high level of satisfaction about the service provided, confirming previous research (32, 33).

Caregivers under the age of 40 showed a higher probability to express that “telerehabilitation made them feel supported during lockdown”; this could be related to the emotional impact of social isolation on this age group (34–37).

Other important personal characteristics of the caregiver group may have influenced the perception of telerehabilitation, such as the severity of the child's disability, the presence of siblings and their age, and if caregivers were working remotely or in the workplace. The analysis of these additional factors could provide more elements for the interpretation of caregivers' perception of telerehabilitation.

CSQ-8 results of caregivers' sample showed a high level of satisfaction with the telerehabilitation service provided, and this finding is in line with data reported in the literature (26, 33, 38). These data do not refer to the level of satisfaction in telerehabilitation itself (3).

### Perception and Satisfaction of Telerehabilitation by Adult Patients

Compared to pediatric patients, adherence rate of adults to telerehabilitation was lower (8.14 and 52.17%, respectively). This can be due both to the greater level of skepticism and to the frequent need of a not-easy-to-find caregiver's assistance to execute the requested exercises. Skepticism about the potential efficacy of telerehabilitation in promoting improvement and goal enhancement has been reported elsewhere in the literature, and it could explain reluctance to consider telerehabilitation as a replacement of face-to-face therapy (39, 40). This reluctance may also be related to the lack of knowledge and experience of this practice, despite the fact that telerehabilitation has shown its efficacy on motor, speech, and cognitive outcomes of adults with neurological disabilities, according to the recent review by Maresca et al. (41–44).

These observations suggest that telerehabilitation should not be generalized. Before proposing this method, its pros and cons, its acceptance, technological resources, confidence with remote media, need and availability of a caregiver's assistance, stress level, and compliance should be considered (45).

Due to the small sample size, a multivariate analysis could not be carried out; thus, the results of this sample's perception and satisfaction with telerehabilitation should be interpreted only as hypotheses.

As for the caregivers' group, adults showed a high level of satisfaction, in line with data reported in previous studies on videoconferencing-delivered interventions (46–49).

### Perception of Rehabilitation Professionals

Professionals also expressed a medium-high level of telerehabilitation perception.

Specialists' answers showed that telerehabilitation allowed them to use their professional abilities for offering support, continuity of care, and a safe environment during lockdown (39).

Alternating telerehabilitation with face-to-face therapy could guarantee both safety of all stakeholders and continuity of care during the phase following lockdown (15).

Professionals reported a high level of agreement with the statement regarding the potential effect of telerehabilitation in enhancing the sense of competence of patients and caregivers in relation to the disability. This perception, based on a screen-mediated observation during remote treatment, has been assessed in previous studies in which professionals' feedback during sessions has shown to make patients and caregivers proactive, thus empowering their ability to care for their loved ones (23, 49–59). Based on the logit regression, remote media skilled professionals and those below the age of 40 have a higher probability to report that telerehabilitation could favor the patient's/family's sense of competence.

In line with the family-centered model, an integration of in-presence therapy with a self-performed or caregiver-mediated home treatment in telerehabilitation could be suggested.

Only a medium level of agreement with the potential benefit of telerehabilitation in enhancing therapeutic goals was reported by professionals. This may be related to skepticism and concern for their patients' clinical outcome. The lack of adequate training, the sudden activation of a treatment method unknown to most of them, and the effort required for adapting the treatment method may be responsible for the fatigue expressed by professionals (15).

Results show that therapists perceived the lack of physical contact as a fundamental limitation to their work as indicated in other studies (60, 61).

Logit regression analysis showed a significant association between the group of physical, occupational, and neurodevelopmental therapists and a good perception of telerehabilitation as a feasible method for maintaining therapeutic goals. Although data are based only on clinical observations made during video calls and not by formal assessment of therapeutic goals, these results are in line with previous studies (62–66). No significant correlations were observed among the level of agreement with the statements and the group of speech therapists and psychologists.

The different level of confidence with remote media and with remote delivered treatment was found to be significantly related to a different perception of telerehabilitation. Based on the logistic analysis, professionals with previous experience in remote delivered treatment have greater odds to perceive telerehabilitation as useful in addition to traditional therapy while those without experience have greater odds to perceive telerehabilitation as a replacement of traditional therapy only in emergency situations. Specific training and dedicated funds are suggested in order to make this a more feasible approach (4, 67). In line with Maresca et al., studies aimed to assess cost-effectiveness of telerehabilitation should be carried out in order to endorse this practice during and beyond periods of health crisis (41).

## Data Availability Statement

The raw data supporting the conclusions of this article will be made available by the authors, without undue reservation.

## Ethics Statement

The studies involving human participants were reviewed and approved by Independent Ethic Committee of the Research Institute of the Santa Lucia Foundation. Written informed consent to participate in this study was provided by the participants' legal guardian/next of kin.

## Author Contributions

CAs: survey design, interpretation of data, and manuscript writing. CAn: critical writing revision. HC and TG: survey design. PG: contribution to the design. SP: statistical analysis and interpretation of data. DM: survey design, survey creation, and interpretation of data. All authors have approved the final version of the article and contributed to the study design.

## Conflict of Interest

The authors declare that the research was conducted in the absence of any commercial or financial relationships that could be construed as a potential conflict of interest.

## References

[1] Doarn CR Latifi R Poropatich RK Sokolovich N Kosiak D Hostiuc F Development and validation of telemedicine for disaster response: the North Atlantic treaty organization multinational system. Telemed J E health. (2018) 24:657–68. 10.1089/tmj.2017.0237 29297764

[2] Zhao J Zhang Z Guo H Li Y Xue W Ren L. E-health in China: challenges, initial directions, end experience. Telemed J E health. (2010) 16:344–9. 10.1089/tmj.2009.0076 20406121

[3] Larsen DL Attkisson CC Hargreaves WA Nguyen TD. Assessment of client/patient satisfaction: development of a general scale. Eval Program Plann. (1979) 2:197–207. 10.1016/0149-7189(79)90094-6 10245370

[4] Attkisson CC Greenfield K. The ucsf client satisfaction scales: I. The client satisfaction questionnaire-8. In: Maruish M, editor. The Use of Psychological Testing for Treatment Planning and Outcome Assessment. 3rd ed. Mahwah, NJ: Lawrence Erlbaum Associates (2004).

[5] Attkisson CC Greenfield TK The client satisfaction questionnaire (CSQ) scales and the service satisfaction scale-30 (SSS-30). In: Seder LL, Dickey B, editors. Outcome Assessment in Clinical Practice. Baltimore: Williams & Wilkins (1996).

[6] Waterfield J Practical research: a guide for therapists. French S, Reynolds F, Swain J.London: Butterworth Heinemann. (2001) (second edition). Physiother Res Int. (2003) 8:164–5. 10.1002/pri.284

[7] Polit DF Beck CT Owen SV. Is the CVI an acceptable indicator of content validity?Appraisal and recommendations. Res Nurs Health. (2007) 30:459–67. 10.1002/nur.20199 17654487

[8] Lynn MR. Determination and quantification of content validity. Nurs Res. (1986) 35:382–5. 10.1097/00006199-198611000-00017 3640358

[9] Polit DF Beck CT. The content validity index: are you sure you know what's being reported? Critique and recommendations. Res Nurs Health. (2006) 29:489–97. 10.1002/nur.20147 16977646

[10] Matsubara C Green J Astorga LT Daya EL Jervoso HC Gonzaga EM Reliability tests and validation test of the client satisfaction questionnaire (CSQ-8) as an index of satisfaction with childbirth-related care among Filipino women. BMC Pregnancy Childbirth. (2013) 13:235 10.1186/1471-2393-13-235 24341288 PMC3878559

[11] Tsai LLY McNamara RJ Dennis SM Moddel C Alison JA McKenzie DK. Satisfaction and experiences with a supervised home-based real time videoconferencing telerehabilitation exercise program in people with chronic obstructive pulmonary disease (COPD). Int J Telerehabil. (2016) 8:27–38. 10.5195/IJT.2016.6213 28775799 PMC5536727

[12] Dobson AJ Barnett AG An Introduction to Generalized Linear Model. 3rd ed. Boca Raton: Chapman & Hall/CRC (2008).

[13] Anderson DR Burnham KP, Model Selection and Multimodel Inference: a Practical Information-theoretic Approach. 2nd ed. New York, NY: Springer (2002).

[14] Austin PC Merlo J. Intermediate and advanced topics in multilevel logistic regression analysis. Stat Med. (2017) 36:3257–77. 10.1002/sim.7336 28543517 PMC5575471

[15] Prvu Bettger J Thoumi A Marquevich V De Groote W Rizzo Battistella L Imamura M. COVID-19: maintaining essential rehabilitation services across the care continuum. BMJ Glob Health. (2020) 5:e002670. 10.1136/bmjgh-2020-002670 32376777 PMC7228480

[16] Trabacca A Russo L. Covid-19 and child disabilities: whom to protect and how. Eur J Phys Rehabil Med. (2020) 56:372–3 10.23736/S1973-9087.20.06309-1 32329591

[17] Russo L Trabacca A. The ethic of care, disability and rehabilitation during the coronavirus disease 19 pandemic. Pediatr Neurol. (2020) 111:39. 10.1016/j.pediatrneurol.2020.06.006 32951656 PMC7297163

[18] Lemacks J Fowles K Mateus A Thomas K. Insights from parents about caring for a child with birth defects. Int J Environ Res Public Health. (2013) 10:3465–82. 10.3390/ijerph10083465 23965922 PMC3774449

[19] Novak I. Evidence-based diagnosis, health care, and rehabilitation for children with cerebral palsy. J Child Neurol. (2014) 29:1141–56. 10.1177/0883073814535503 24958005

[20] Alaee N Shahboulaghi F Khankeh H Mohammad Khan Kermanshahi S Psychosocial challenges for parents of children with cerebral palsy: a qualitative study. J Child Fam Stud. (2015) 24:2147–54. 10.1007/s10826-014-0016-3

[21] Inglese CN Elliott AM CAUSES Study Lehman A. New developmental syndromes: understanding the family experience. J Genet Couns. (2019) 28:202–12. 10.1002/jgc4.1121 30938469

[22] Beckers LWME Smeets RJEM van der Burg JJW. Therapy-related stress in parents of children with a physical disability: a specific concept within the construct of parental stress. Disabil Rehabil. (2019). 10.1080/09638288.2019.1646815. [Epub ahead of print]. 31424960

[23] Colyvas J Sawyer L Campbell P. Identifying strategies early intervention occupational therapists use to teach caregivers. Am J Occup Ther. (2010) 64:776–85. 10.5014/ajot.2010.09044 21073108

[24] Coyne LW Gould ER Grimaldi M Wilson KG Baffuto G Biglan A. First things first: parent psychological flexibility and self-compassion during COVID-19. Behav Anal Pract. (2020). 10.1007/s40617-020-00435-w. [Epub ahead of print]. 32377315 PMC7200171

[25] Phillips D Paul G Fahy M Dowling-Hetherington L Kroll T Moloney B. The invisible workforce during the COVID-19 pandemic: family carers at the frontline. HRB Open Res. (2020) 3:24. 10.12688/hrbopenres.13059.1 32551415 PMC7276936

[26] Fazzi E Galli J. New clinical needs and strategies for care in children with neurodisability during COVID-19. Dev Med Child Neurol. (2020) 62:879–80. 10.1111/dmcn.14557 32358977 PMC7267576

[27] Ismail FY Fatemi A Johnston MV. Cerebral plasticity: windows of opportunity in the developing brain. Eur J Paediatr Neurol. (2017) 21:23–48. 10.1016/j.ejpn.2016.07.007 27567276

[28] Larson E Miller-Bishoff T. Family routines within the ecological niche: an analysis of the psychological well-being of U.S. caregivers of children with disabilities. Front Psychol. (2014) 5:495. 10.3389/fpsyg.2014.00495 24910625 PMC4038926

[29] Imran N Zeshan M Pervaiz Z. Mental health considerations for children & adolescents in COVID-19 pandemic. Pak J Med Sci. (2020) 36:S67–72. 10.12669/pjms.36.COVID19-S4.2759 32582317 PMC7306970

[30] Knox V Do parents of children with cerebral palsy express different concerns in relation to their child's type of cerebral palsy, age and level of disability? Physiotherapy. (2008) 94:56–62. 10.1016/j.physio.2007.04.005

[31] Beresford B Clarke S Maddison J. Therapy interventions for children with neurodisabilities: a qualitative scoping study. Health Technol Assess. (2018) 22:1–150. 10.3310/hta22030 29345224 PMC5787698

[32] Ray KN Ashcraft LE Mehrotra A Miller E Kahn JM. Family perspectives on telemedicine for pediatric subspecialty care. Telemed J E Health. (2017) 23:852–62. 10.1089/tmj.2016.0236 28430021 PMC5651976

[33] Camden C Pratte G Fallon F Couture M Berbari J Tousignant M. Diversity of practices in telerehabilitation for children with disabilities and effective intervention characteristics: results from a systematic review. Disabil Rehabil. (2019) 42:3424–36. 10.1080/09638288.2019.1595750 30978110

[34] Sim K Huak Chan Y Chong PN Chua HC Wen Soon S. Psychosocial and coping responses within the community health care setting towards a national outbreak of an infectious disease. J Psychosom Res. (2010) 68:195–202. 10.1016/j.jpsychores.2009.04.004 20105703 PMC7094450

[35] Taylor M Agho KE Stevens G Raphael B. Factors influencing psychological distress during a disease epidemic: data from Australia's first outbreak of equine influenza. BMC Public Health. (2008) 8:347. 10.1186/1471-2458-8-347 18831770 PMC2571100

[36] Dubey S Biswas P Ghosh R Chatterjee S Dubey MJ Chatterjee S Psychosocial impact of COVID-19. Diabetes Metab Syndr. (2020) 14:779–88. 10.1016/j.dsx.2020.05.035 32526627 PMC7255207

[37] Gómez-Salgado J Andrés-Villas M Domínguez-Salas S Díaz-Milanés D Ruiz-Frutos C. Related health factors of psychological distress during the COVID-19 pandemic in Spain. Int J Environ Res Public Health. (2020) 17:3947. 10.3390/ijerph17113947 32498401 PMC7312369

[38] Bird M Li L Ouellette C Hopkins K McGillion MH Carter N. Use of synchronous digital health technologies for the care of children with special health care needs and their families: scoping review. JMIR Pediatr Parent. (2019) 2:e15106. 10.2196/15106 31750840 PMC6895870

[39] McIntyre M Robinson LR Mayo A. Practical considerations for implementing virtual care in physical medicine and rehabilitation: for the pandemic and beyond. Am J Phys Med Rehabil. (2020) 99:464–7. 10.1097/PHM.0000000000001453 32324617 PMC7253038

[40] Peretti A Amenta F Tayebati SK Nittari G Mahdi SS. Telerehabilitation: review of the state-of-the-art and areas of application. JMIR Rehabil Assist Technol. (2017) 4:e7. 10.2196/rehab.7511 28733271 PMC5544892

[41] Maresca G Maggio MG De Luca R Manuli A Tonin P Pignolo L. Tele-neuro-rehabilitation in Italy: state of the art and future perspectives. Front Neurol. (2020) 11:563375. 10.3389/fneur.2020.563375 33101176 PMC7554582

[42] Maresca G Maggio MG Latella D Cannavò A De Cola MC Portaro S. Toward improving poststroke aphasia: a pilot study on the growing use of telerehabilitation for the continuity of care. J Stroke Cerebrovasc Dis. (2019) 28:104303. 10.1016/j.jstrokecerebrovasdis.2019.104303 31371144

[43] Tinelli F Cioni G Purpura G. Development and implementation of a new telerehabilitation system for audiovisual stimulation training in hemianopia. Front Neurol. (2017) 8:621. 10.3389/fneur.2017.00621 29209271 PMC5702450

[44] Torrisi M Maresca G De Cola MC Cannavò A Sciarrone F Silvestri G. Using telerehabilitation to improve cognitive function in post-stroke survivors: is this the time for the continuity of care? Int J Rehabil Res. (2019) 42:344–51. 10.1097/MRR.0000000000000369 31464812

[45] Klaic Marlena Galea Mary P. Using the technology acceptance model to identify factors that predict likelihood to adopt tele-neurorehabilitation. Front Neurol. (2020) 11:1637. 10.3389/fneur.2020.580832 33343488 PMC7738474

[46] Cranen K Drossaert CH Brinkman ES Braakman-Jansen AL Ijzerman MJ Vollenbroek-Hutten MM. An exploration of chronic pain patients' perceptions of home telerehabilitation services. Health Expect. (2012) 15:339–50. 10.1111/j.1369-7625.2011.00668.x 21348905 PMC5060638

[47] Steel K Cox D Garry H. Therapeutic videoconferencing interventions for the treatment of long-term conditions. J Telemed Telecare. (2011) 17:109–17. 10.1258/jtt.2010.100318 21339304

[48] Kairy D Lehoux P Vincent C Visintin M. A systematic review of clinical outcomes, clinical process, healthcare utilization and costs associated with telerehabilitation. Disabil Rehabil. (2009) 31:427–47. 10.1080/09638280802062553 18720118

[49] Cranen K Groothuis-Oudshoorn CG Vollenbroek-Hutten MM IJzerman MJ. Toward patient-centered telerehabilitation design: understanding chronic pain patients' preferences for web-based exercise telerehabilitation using a discrete choice experiment. J Med Internet Res. (2017) 19:e26. 10.2196/jmir.5951 28108429 PMC5291864

[50] Sawyer BE Campbell PH Early interventionists' perspectives on teaching caregivers. J Early Interv. (2012) 34:104–24. 10.1177/1053815112455363

[51] Jansen LM Ketelaar M Vermeer A. Parental experience of participation in physical therapy for children with physical disabilities. Dev Med Child Neurol. (2003) 45:58–69. 10.1111/j.1469-8749.2003.tb00861.x 12549757

[52] Baker T Haines S Yost J DiClaudio S Braun C Holt S The role of family-centered therapy when used with physical or occupational therapy in children with congenital or acquired disorders. Phys Ther Rev. (2012) 17:29–36. 10.1179/1743288X11Y.0000000049

[53] Hall N Boisvert M Steele R. Telepractice in the assessment and treatment of individuals with aphasia: a systematic review. Int J Tele ehabil. (2013) 5:27–38. 10.5195/IJT.2013.6119 25945211 PMC4296832

[54] Appleby E Gill ST Hayes LK Walker TL Walsh M Kumar S. Effectiveness of telerehabilitation in the management of adults with stroke: A systematic review. PLoS ONE. 14:e0225150. 10.1371/journal.pone.0225150 31714924 PMC6850545

[55] Chen Y Abel KT Janecek JT Chen Y Zheng K Cramer SC. Home-based technologies for stroke rehabilitation: a systematic review. Int J Med Inform. (2019) 123:11–22. 10.1016/j.ijmedinf.2018.12.001 30654899 PMC6814146

[56] Sarfo FS Ulasavets U Opare-Sem OK Ovbiagele B. Tele-rehabilitation after stroke: an updated systematic review of the literature. J Stroke Cerebrovasc Dis. (2018) 27:2306–18. 10.1016/j.jstrokecerebrovasdis.2018.05.013 29880211 PMC6087671

[57] Calabrò RS Bramanti A Garzon M Celesti A Russo M Portaro S. Telerehabilitation in individuals with severe acquired brain injury: rationale, study design, and methodology. Medicine. (2018) 97:e13292. 10.1097/MD.0000000000013292 30557976 PMC6320067

[58] Ceravolo MG de Sire A Andrenelli E Negrini F Negrini S Systematic rapid “living” review on rehabilitation needs due to covid-19: update to march 31st 2020. Eur J Phys Rehabil Med. (2020) 56:347–53. 10.23736/S1973-9087.20.06329-7 32316718

[59] de Sire A Andrenelli E Negrini F Negrini S Ceravolo MG. Systematic rapid “living” review on rehabilitation needs due to COVID-19: update as of April 30th 2020. Eur J Phys Rehabil Med. (2020) 56:354–60. 10.23736/S1973-9087.20.06378-9 32408729

[60] Lawford BJ Bennell KL Kasza J Hinman RS. Physical therapists' perceptions of telephone- and internet video-mediated service models for exercise management of people with osteoarthritis. Arthritis Care Res. (2018) 70:398–408. 10.1002/acr.23260 28437566

[61] Grona SL Bath B Busch A Rotter T Trask C Harrison E. Use of videoconferencing for physical therapy in people with musculoskeletal conditions: a systematic review. J Telemed Telecare. (2018) 24:341–55. 10.1177/1357633X17700781 28403669

[62] Benfer KA Novak I Morgan C Whittingham K Khan NZ Ware RS. Community-based parent-delivered early detection and intervention programme for infants at high risk of cerebral palsy in a low-resource country (Learning through everyday activities with parents (LEAP-CP): protocol for a randomised controlled trial. BMJ Open. (2018) 8:e021186. 10.1136/bmjopen-2017-021186 29934387 PMC6020941

[63] Lee MJ Yoon S Kang JJ Kim J Kim JM Han JY. Efficacy and safety of caregiver-mediated exercise in post-stroke rehabilitation. Ann Rehabil Med. (2018) 42:406–15. 10.5535/arm.2018.42.3.406 29961738 PMC6058591

[64] Lillo-Navarro C Montilla-Herrador J Escolar-Reina P Oliveira-Sousa SL García-Vidal JA Medina-Mirapeix F. Factors associated with parents' adherence to different types of exercises in home programs for children with disabilities. J Clin Med. (2019) 8:456. 10.3390/jcm8040456 30959749 PMC6518115

[65] Cottrell MA Galea OA O'Leary SP Hill AJ Russell TG. Real-time telerehabilitation for the treatment of musculoskeletal conditions is effective and comparable to standard practice: a systematic review and meta-analysis. Clin Rehabil. (2017) 31:625–38. 10.1177/0269215516645148 27141087

[66] Hung Kn G Fong KN. Effects of telerehabilitation in occupational therapy practice: a systematic review. Hong Kong J Occup Ther. (2019) 32:3–21. 10.1177/1569186119849119 31217758 PMC6560836

[67] Dorsey ER Topol EJ State of telehealth. N Engl J Med. (2016) 375:154–61. 10.1056/NEJMra1601705 27410924

